# Towards the diagnosis of osteoporosis – contributions from coincidental diagnostic imaging findings in chiropractors' practice

**DOI:** 10.1186/s12998-024-00545-0

**Published:** 2024-06-24

**Authors:** Lars Uhrenholt, Jakob Hermannsen Bakkegaard, Kasper Hansen, Klaus Knarberg Doktor

**Affiliations:** 1 Department of Forensic Medicine, Aarhus University, Palle Juul-Jensens Boulevard 99, 8200 Aarhus N, Denmark; 2 Nortvig & Uhrenholt Kiropraktisk Klinik, Jens Baggesens Vej 88A, 8200 Aarhus N, Denmark; 3 Chiropractic Knowledge Hub, University of Southern Denmark, Campusvej 55, 5230 Odense M, Denmark; 4 Rygcenter Midtvest, Dalgas Alle 2, 7400 Herning, Denmark

**Keywords:** Osteoporosis, Opportunistic approach, Chiropractic practice, Radiological examination, Early diagnosis

## Abstract

**Background:**

Osteoporosis is significantly associated with fractures and burdens the health of especially older people. Osteoporotic fractures cause pain, disability, and increased mortality. Early diagnosis of osteoporosis allows earlier initiation of treatment, thereby reducing the risk of osteoporotic fractures. Chiropractors encounter potential osteoporotic patients daily, and perform radiological evaluation of these and other patients, including evaluation of X-rays done for other purposes than osteoporosis. Therefore, chiropractors may identify vertebral fractures, vertebral deformity or osteopenia not otherwise suspected or recorded.

**Methods:**

This study examines procedures available to the chiropractor to describe conventional X-rays with the focus of osteoporosis related findings. We review the indications for radiological examination in chiropractic practice, and in the realm of osteoporosis we describe radiological methods available for examination of conventional radiographs, and the necessity of inter-disciplinary communication.

**Results:**

National guidelines are available regarding referral for X-rays in chiropractic practice. Standardized protocols ensure image acquisition of the highest quality in the chiropractors’ radiological department. Conventional X-ray examination is not indicated on clinical suspicion of osteoporosis alone, as bone mineral density testing is the diagnostic test. Radiological assessment of all available X-rays of patients above the age of 50 years should include evaluation of the bone quality, and hip and vertebral fracture assessment. The Singh index, Genant Semi-Quantitative tool (GSQ), and Algorithm-Based Qualitative method (ABQ) should be used consistently during interpretation. Referral for additional imaging and evaluation should be prompt and systematic when needed.

**Conclusions:**

This article presents an overview of evidence-based radiological procedures for the purpose of promoting early diagnosis of osteoporosis. We present recommendations to the clinicians where we propose an opportunistic evaluation of X-rays, done for any reason, which include systematic evaluation of bone quality, presence of hip and vertebral fractures, and vertebral deformation of all patients above the age of 50 years. Detailed referral to healthcare professionals for further diagnostic evaluation is performed when needed. Consistent, high-quality radiological procedures in chiropractic practices could feasibly contribute to the timely diagnosis of osteoporosis, ultimately minimizing the impact of osteoporosis-related complications on patients’ health.

## Background

Osteoporosis is a common condition affecting approximately 660.000 Danes over the age of 50 years, equivalent to 29% of the population in this age group [1]. Similarly, a recent report from the National Center for Health Statistics in the United States estimated 12.6% of the population above the age of 50 years to have osteoporosis, with a higher prevalence among women compared with men [2]. Hence, there is considerable variation in prevalence between countries and regions [3, 4]. Furthermore, it is generally acknowledged that a large percentage of these patients are undiagnosed with osteoporosis, e.g. approximately 66% of the Danish population [1, 5]. Osteoporosis is characterized by reduced bone mass and deterioration of bone quality and is complicated by fractures that become increasingly common in the aging osteoporotic skeleton [6, 7]. It is estimated that 50% of women and 20% of men over the age of 50 years will suffer an osteoporosis-related fracture during the remainder of their life [4, 6]. These fractures are associated with pain, reduced physical performance, disability, significant postural changes and early death [7–9]. However, in the vast majority of cases, osteoporosis does not cause any identifiable clinical signs or symptoms before fractures occur. Hence, as the clinical manifestations of osteoporosis are the results of fractures, preventive measures, early diagnosis, and relevant treatment is warranted to limit the detrimental effects of the disease, preferably before the first fracture occurs [7].

**Fig. 1:**
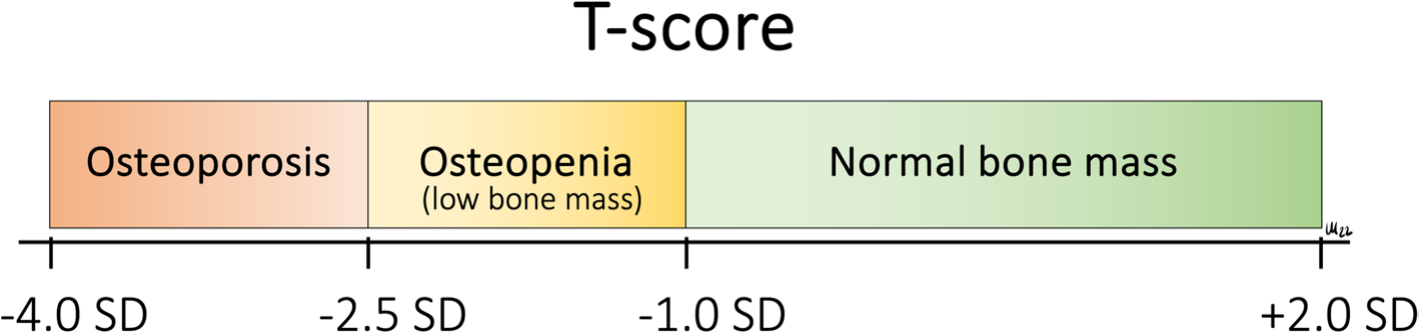
Classification of bone mineral density (BMD) based on the T-score. The T-score describes the deviation in bone mineral density (BMD) from the BMD-value of a healthy young individual of the same sex and ethnicity (standard reference) as measured in standard deviations (SD)

According to the World Health Organization (WHO), osteoporosis is defined as a condition in which the bone mineral density (BMD, in units of mg/cm^3^) is 2.5 standard deviations (SD) or more below the average value for young, healthy individuals (i.e. a T-score ≤ -2.5) in the lumbar spine or the hip (Fig. 1) [4]. The BMD measurement used for diagnosis of osteoporosis is usually based on bone densitometry, which conveniently can be obtained non-invasively using Dual-energy X-ray Absorptiometry (DEXA) scanning [10]. The T-score is a dimensionless unit where a patient’s BMD measurement is compared to the average BMD of young individuals of the same sex and ethnicity, with the difference expressed in SD of the distribution around the mean of the reference group [4]. In some cases, a Z-score, which also considers the age of the patient, is calculated. The T-score and Z-score are tools exploited to evaluate a patient’s likely progression towards osteoporosis based on a BMD-value obtained, e.g. using DEXA [4].

Chiropractors encounter potential osteoporotic patients on a daily basis. Clinical observations and information from the case history may point towards the diagnosis. Risk factors include for example, family history of osteoporosis, endocrine disorders, increasing age, long-term use of certain medications such as corticosteroids, alcohol abuse, and low body mass index (BMI) [7]. In addition, findings from radiographic procedures may raise suspicion of osteoporosis. These include, for example, the presence of a range of alterations in the appearance of the vertebrae, from subtle signs of osteopenia to well-defined vertebral fractures, hip fractures, or more general osteopenic appearance of the skeleton. For all new patients and new complaints, the chiropractor undertakes an evaluation as to whether a radiological examination should be performed or not. According to international guidelines, the indicators for radiological examination are many, as will be described later in the main text. These include for example age above 50 years, poor improvement with conservative care, trauma, history of fracture, drug and alcohol abuse [11–14]. It is important to note, however, that none of the indicators alone would indicate the need for imaging.

In Denmark, chiropractors utilize diagnostic imaging including X-rays, CT-scanning, Magnetic Resonance Imaging (MRI) and Ultrasonography in 23.1% of all new patients [15]. The majority of the examinations consist of in-house X-rays primarily performed of new patients. Under the assumption that new patients received their X-ray examination in relation to their first presentation in the clinic it can be estimated, based on statistics from the Danish Region annual statements of services provided by Danish chiropractors, that X-rays were used in 18.4% of cases in 2023 [15].

A large number of the patients who are examined with X-rays will be above the age of 50 years, which in the context of osteoporosis is particularly relevant, as they have a higher risk of having the disease.

Hence, in some cases, the chiropractors will evaluate X-ray images that, most often by coincidence, show signs of osteopenia and/or osteoporotic fractures in the absence of a diagnosis or suspicion of osteoporosis. The inherent possibility of identifying these signs of osteoporosis on conventional X-rays, irrespective that the reasons for obtaining the images, provides a unique opportunistic opportunity, which given the chiropractor's awareness, could likely contribute to earlier diagnosis of osteoporosis in some individuals [7, 16–21].

We present an overview of evidence-based radiological procedures and provide recommendations that enables the clinician to produce radiological reports of relevance to the general practitioner (GP) and specialized hospital departments.

## Main text

### Diagnostic imaging in chiropractic settings

On clinical indication, the chiropractor performs a conventional radiological examination of a variety of conditions covering the entire skeleton. International guidelines for the indication of spinal radiography are available, including listing of indicators that must be used in conjunction with other clinical indicators derived from the case history and clinical examination [11–14]. For chiropractors, who generally utilize high-velocity low-amplitude spinal manipulative therapy (HVLA-SMT), as the most common intervention to their patients (79% of all patients) [22], radiological examination can be used, based on relevant clinical indications, as an assessment tool in order to ensure that there are no contraindications to HVLA-SMT [12–14]. It is not recommended that chiropractors (or any other healthcare professional) take routine X-rays of all patients [7, 17].

As asymptomatic vertebral fractures are increasingly common in aging elderly patients, particularly radiological assessment of the integrity of the spinal column, e.g. vertebral fracture assessment (VFA) should be considered of the high-risk individuals listed in Table 1 [7, 21, 23]. The VFA is historically performed using conventional lateral thoracic and lumbar X-rays, with DEXA-VFA emerging as a novel alternative according to the American College of Radiology [10, 24]. It is important to note, that recently obtained images acquired for other purposes than osteoporosis can and should be used for VFA [7, 18, 21], including relevant X-rays obtained in the chiropractors’ radiological departments [7, 23]. One could reasonably argue that chiropractors, who consider treating these elderly high-risk patients (as listed in Table 1), potentially including HVLA-SMT, in most cases should ensure that a VFA is performed prior to initiation of treatment.

**Table 1:** High-risk patients—Indications for radiological Vertebral Fracture Assessment

• All women aged ≥ 65 years and all men aged ≥ 80 years, T-sore ≤ -1.0 (lumbar spine, total hip, femoral neck)
• Men aged 70–79 years, T-score ≤ -1.5 (lumbar spine, total hip, femoral neck)
• Postmenopausal women and men age ≥ 50 years with specific risk factors:
- Fractures during adulthood (age ≥ 50 years)
- Historical height loss of 3.8 cm or more*
- Prospective height loss of 2.0 cm or more**
- Recent or ongoing long-term glucocorticoid treatment
- Medical conditions associated with bone loss such as hyperparathyroidism

*NB* If bone density testing is not available, vertebral imaging may be considered based on age alone

^*^Current height compared to peak height during young adulthood

^**^Cumulative height loss measured during interval medical assessment

Adapted from: Leboff et al. 2022 [7]

In the context of osteoporosis, X-rays of the skeleton, particularly the lumbar spine, pelvis, and hip joints of patients 50 years of age and above, are all potentially relevant for the early diagnosis of osteoporosis [7, 17].

### Radiographic procedures

There are standardized protocols for obtaining radiological images of the skeleton, including the lumbar spine, pelvis, and hip joint, although with some variations between countries [25, 26]. The chiropractor should always adhere to these protocols to obtain images of highest quality, i.e. correct positioning, collimation, and exposure, and always adhering to the as-low-as-reasonably-achievable (ALARA) principle. Protective lead shielding should be applied when relevant.

### The radiological assessment

Examination of radiological images is no simple task and requires a combination of knowledge, skill, and experience. As these parameters are susceptible to some variations, the European Society of Radiology has published a framework for radiological practice [27]. The benefit of adherence to these suggestions is a high-quality radiological report with the highest relevance as a medical document for prospective care planning. In the context of osteoporosis, the chiropractor should evaluate all X-rays of particularly the lumbar spine, pelvis, and hip joints of patients aged 50 years and above, irrespective of the indication prompting the acquisition of the X-rays, using the systematic methods described below. Whenever osteoporosis related radiological findings are suspected or confirmed, e.g. osteopenia, vertebral fracture, or hip fracture, the radiological report produced by the chiropractor should include a description of the findings and a conclusive diagnosis. This should be forwarded to the GP and/or specialized facilities with a referral for additional diagnostic evaluation. If a vertebral fracture is identified, the term “vertebral fracture” should always be used [17].

### Assessment of the bone quality

**Fig. 2:**
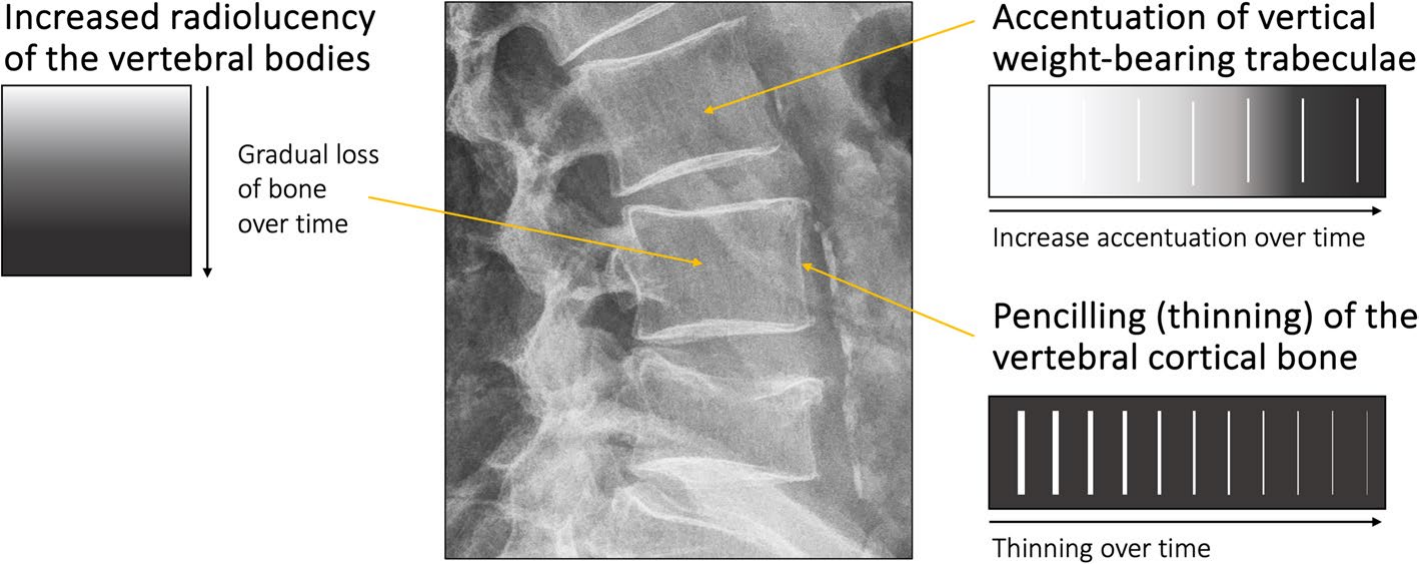
Evaluation of osteopenia in the spinal vertebrae. This figure illustrates frequent signs of osteopenia visible on conventional standing lumbar spine lateral X-ray, e.g. pencilling/thinning of the vertebral cortex, generalized increased radiolucency of the vertebral bodies, and accentuation of the vertically oriented trabeculae

The bone quality is routinely assessed during conventional radiological examination of the lumbar spine, pelvis, and/or hip joints. Albeit a subjective evaluation, and with some limitations, radiological evaluation of the bone quality has been shown to be a strong predictor of reduced bone quality, i.e. osteopenia or osteoporosis [28–30]. When the skeleton shows signs of reduced bone quality, e.g. osteopenia (Fig. 2), a DEXA-scan is warranted to determine the actual BMD [10]. On the examination of conventional X-rays, critical features of reduced bone quality in the spine include the radiolucent appearance of the trabecular bone, which often displays accentuation of the vertical trabeculae (striated pattern, as displayed in Fig. 2). This results from more significant loss of horizontal trabeculae, i.e. the non-weight bearing part, than vertical trabeculae [31]. In addition, the cortical bone becomes thinned with a more distinct outline of the bone, also known as pencilling. However, the absence of osteopenia on a conventional radiological examination does not preclude osteoporosis. In recent years, the combination of technical advancements of conventional radiographic equipment and developing artificial intelligence (AI) models, e.g. deep learning models through deep convolutional neural networks (DCNN), has shown that screening conventional X-rays for bone quality is both relevant and valuable in the context of osteoporosis [18–20].

**Fig. 3:**
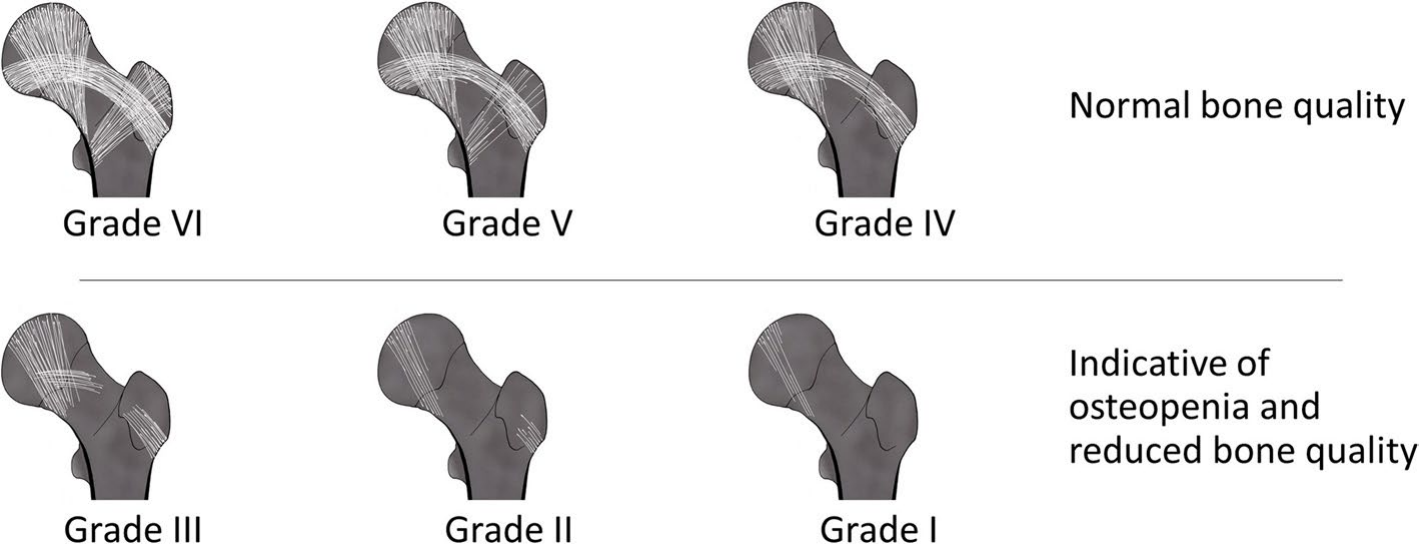
Evaluation of osteopenia in the hip using the Singh index. The Singh index can be applied according to the following grades, where Grade I-III is indicative of osteopenia; Grade VI: All the trabecular subgroups are visible. Grade V: The primary tensile and compressive trabeculae are slightly reduced, and the Ward triangle remains prominent. Grade IV: The primary tensile trabeculae are significantly reduced, but continuous from the lateral cortex to the femoral neck. Grade III: The primary tensile trabeculae are deficient/interrupted. Grade II: Only the primary compressive trabeculae are present albeit reduced. Grade I: The remaining primary compressive trabeculae are severely reduced. The figure is adapted from Alabdah et al*. *[33]

Similar to the changes described in the spinal column, loss of non-weight bearing bone in the proximal femur will accentuate the curved dome-shaped trabecular architecture in the remaining weight-loaded trabeculae. The Singh index (SI) (Fig. 3) is a known measure of osteopenia in the femur [32, 33]. Although SI suffers from inaccurate estimates, i.e. low sensitivity, it has a high specificity which allows its use as an indicator of osteopenia [32, 34, 35]. However, this index cannot stand alone and should be utilized in combination with other tools such as DEXA when diagnosing osteoporosis [33].

### The Genant Semi-Quantitative tool (GSQ)

**Fig. 4:**
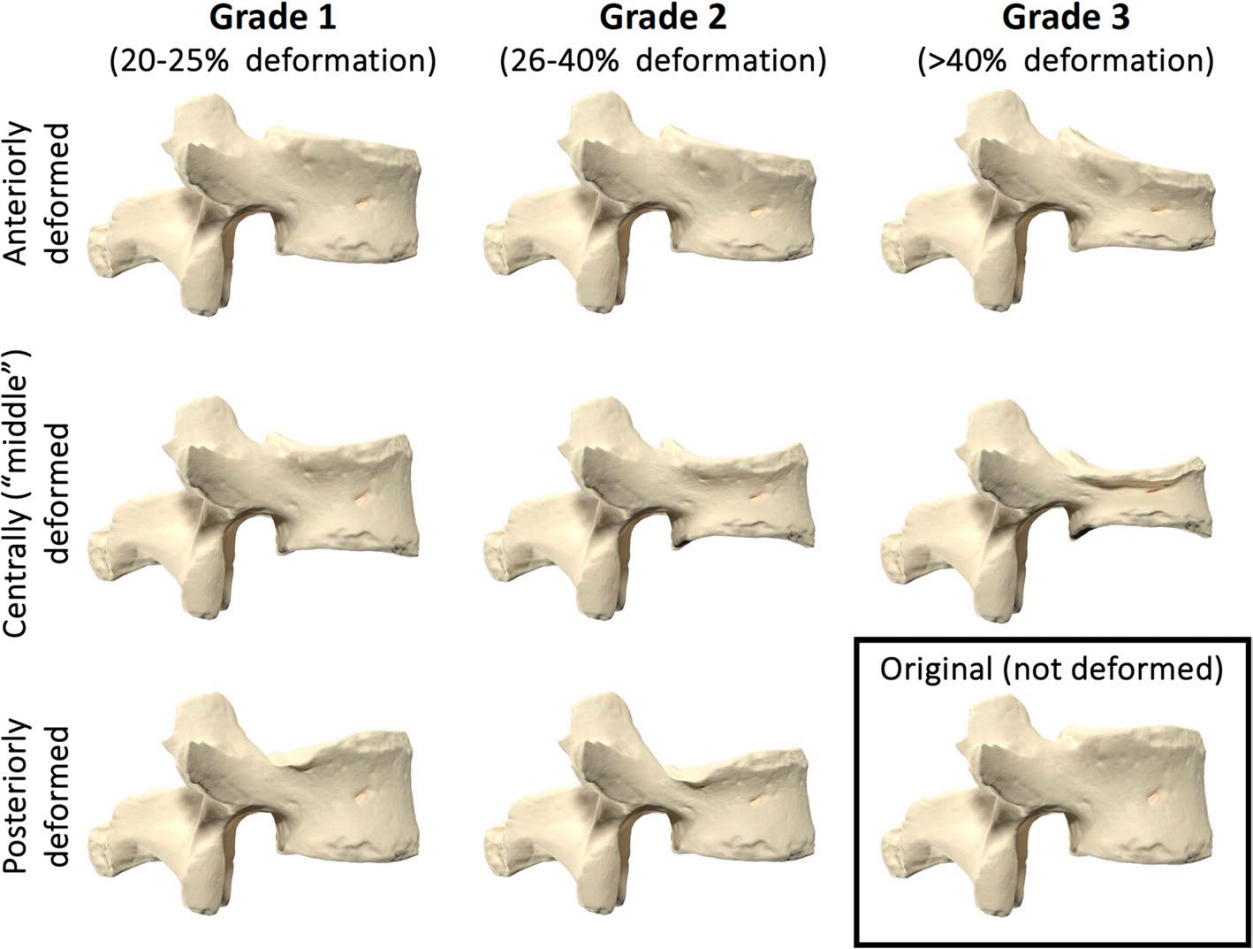
Genant Semi-Quantitative tool (GSQ). This figure illustrates the Genant Semi-Quantitative tool (GSQ) using a 3D model of a lumbar vertebrae (original in the lower right corner), which were modified using 3D software to visualize the three grades of vertebral body compression anteriorly, centrally (“middle”), and posteriorly. Figure adapted from Lentle et al. [17] and Genant et al. [36]. The original vertebral model was acquired from the gallery of Eric Bauer and the donor-based undergraduate human anatomy lab at Elon University, North Carolina, USA (released under the Attribution 4.0 International (CC BY 4.0) license; link to source file:* “*https://commons.wikimedia.org/wiki/File:Human_lumbar_vertebra.stl*”. *All mesh-modifications and renders were made using the freeware software “Blender” (Blender Development Team (2023). Blender (Version 3.5.1),* “*https://www.blender.org*”*

**Fig. 5:**
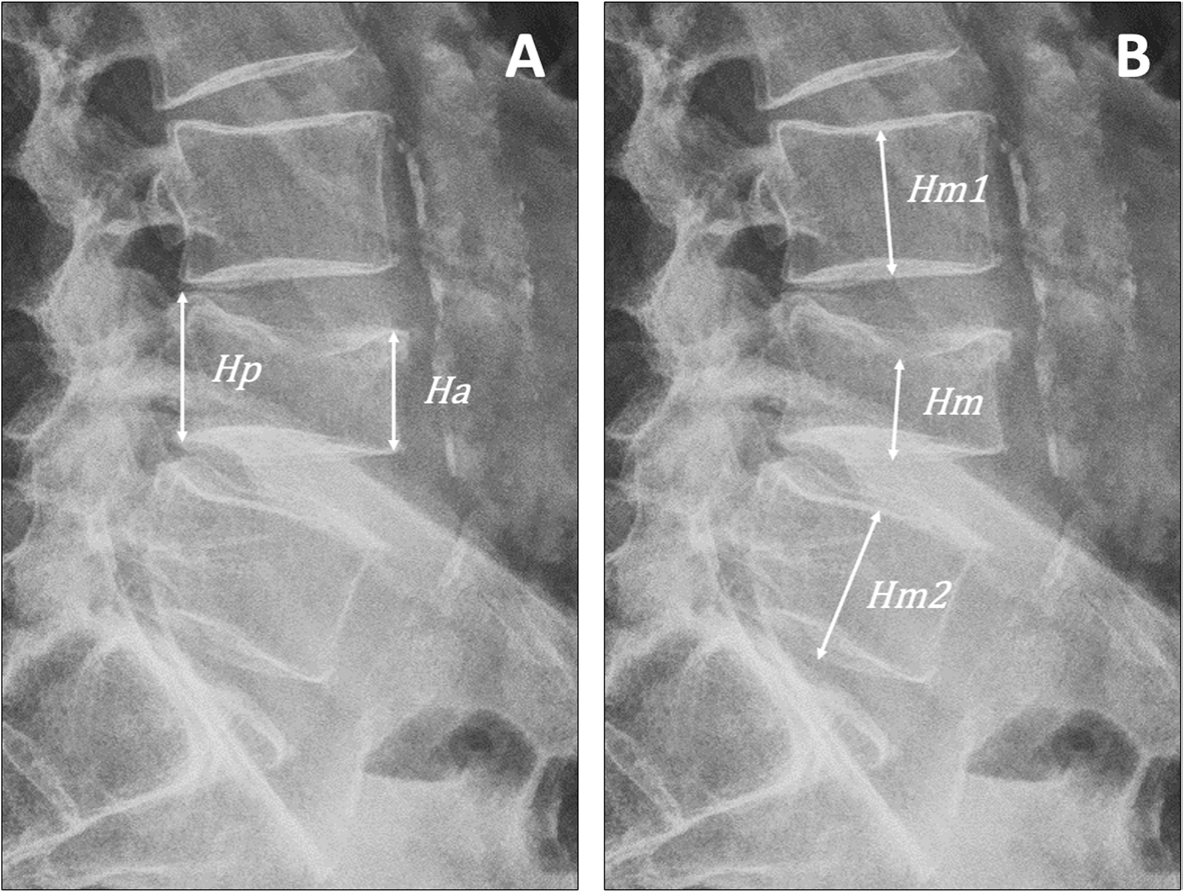
Calculation of vertebral body height reduction. A 76-year-old male consulting a chiropractic clinic with acute low back pain of four days duration following an episode of lifting a heavy garden pot. An in-house standing X-ray examination of the lumbar spine was performed showing a new vertebral compression fracture of L4. This figure is a closeup of the lateral projection, which illustrates a practical procedure to calculate the percentage of vertebral body height reduction of an involved vertebrae*.* **A** Anterior height reduction $\left(\mathbf{\%}\right)=\frac{Hp -Ha}{Hp}*100$. **B** To calculate the middle height reduction of a vertebral body, the height is compared to the mean of the two neighbouring vertebral bodies*; *$m\left(Hm1,Hm2\right)=\frac{Hm1+Hm2}2$. Middle height reduction $\left(\mathbf{\%}\right)=\frac{m\left(Hm1, Hm2\right) -Hm}{m(Hm1, Hm2)}*100$

Deviations from the normal vertebral morphometry are suspicious when describing the integrity of the spinal column. The Genant Semi-Quantitative tool (GSQ) is a visual method for describing quantitative and qualitative characteristics of the vertebral body and has proven useful as a reliable tool for radiological interpretation and diagnosis of vertebral fractures [17, 21, 36]. GSQ is particularly known for its illustrative charts of graded deformation (Fig. 4) but also describes how to identify end-plate fractures [17, 36]. The anterior (Ha), middle (Hm), and posterior (Hp) vertebral vertical dimensions (i.e. their heights) are measured to allow more precise estimates of height reduction and grading (Fig. 5). The diagnostic criteria for a vertebral fracture, according to this model, is a reduction in Ha, Hm, or Hp height of at least 20% [17, 36].

### The Algorithm-Based Qualitative method

**Fig. 6:**
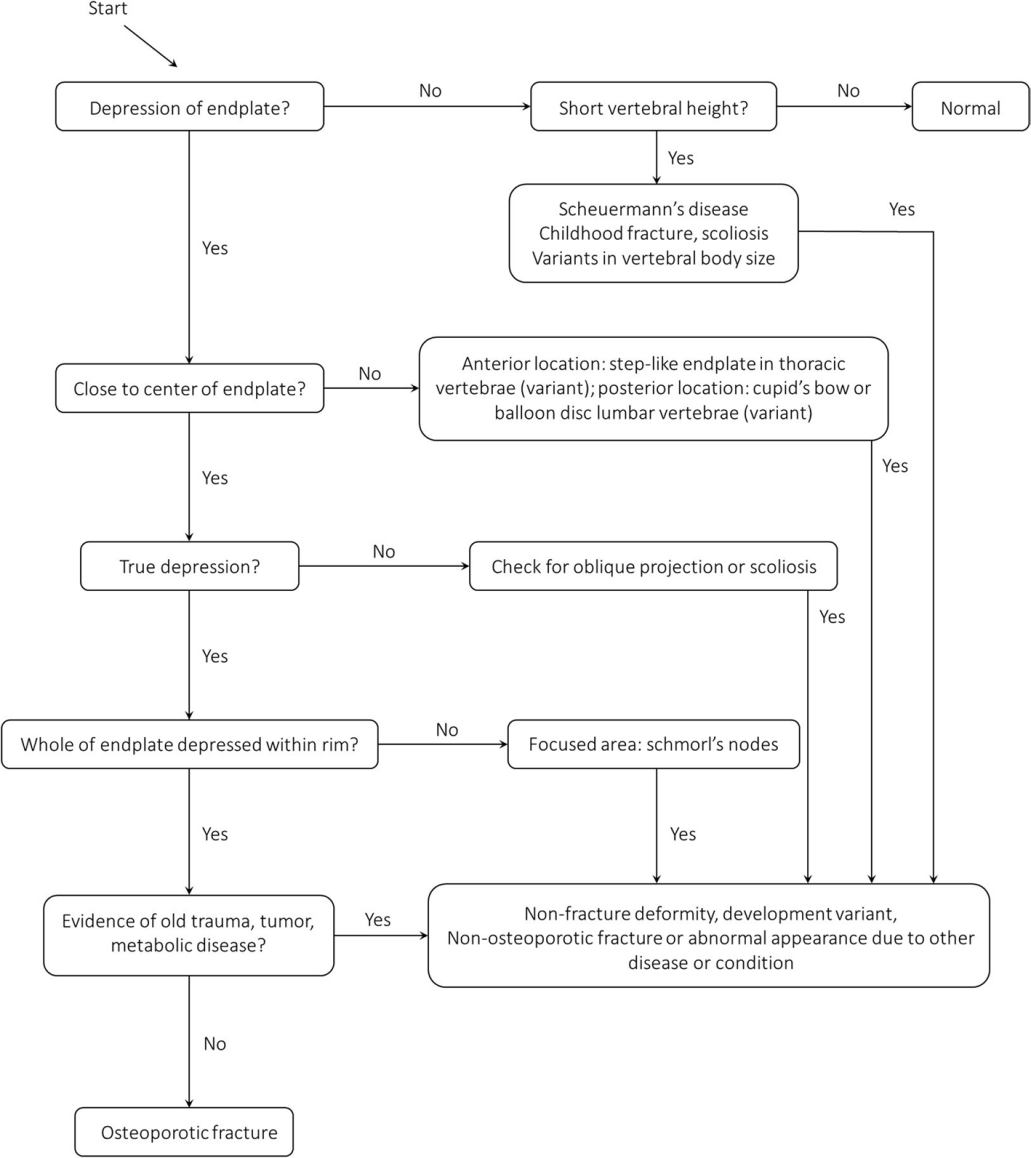
Flow diagram of algorithm-based qualitative (ABQ) identification of vertebral fracture. Adapted from Wang et al., 2017 [38]

The algorithm-based qualitative method (ABQ) was originally described by Jiang et al. for the purpose of identifying osteoporotic vertebral fractures (OVF) [37]. The ABQ differs from GSQ by focusing on endplate depression/fracture instead of vertebral height reduction [37]. Thus, ABQ classifies OVF without measurable changes in vertebral height as it “only” requires evidence of endplate involvement (i.e. depression) [37–39]. The ABQ will systematically exclude non-fracture vertebral deformity that is not related to osteoporotic fractures, for example wedged vertebrae in Scheuermann’s disease (Fig. 6).

### Estimation of the age of a vertebral fracture

A common challenge in clinical practice is the estimation of the age of a vertebral fracture, i.e. a new versus older fracture. Thorough inspection of the conventional X-rays often allows estimation of fracture age, however, in some cases a magnetic resonance imaging (MRI) examination is needed [40–42]. Table 2 presents several features that can assist in the estimation of a fracture’s age. Older vertebral fractures usually show signs of remodeling and associated degenerative changes, the absence of step defects, and on MRI also a recovery of normal bone marrow signal. On the contrary, new/recent vertebral fractures are characterized by a step defect, a zone of impaction, callus formation (which is usually resorbed over time), and on MRI also subchondral edema/bone marrow edema [40, 41]. As a diagnostic challenge, insufficiency fractures associated with osteoporosis (i.e. OVF) very often have no visible step defect, fracture line or callus, and the morphometry remains unchanged irrespective of the age of the fracture. In these cases, MRI is specifically relevant to determine old versus new fracture, if necessary (Table 2).

**Table 2:** Radiological interpretation of new versus old vertebral fracture

Findings	New fracture	Old fracture
**Morphometry**	Altered, e.g. wedge shape and/or depression in endplate	Altered, e.g. wedge shape and/or depression in endplate
**Fracture line**	Present, may be difficult to identify	Absent, be aware of incomplete healing with pseudo-joint
**Step-defect**	Present, with characteristic demarcation of edges	Absent, healed step-defects have smoothened edges
**Zone of impaction**	Present, reactive sclerosis in proximity to the fracture site	Absent, sclerosis of endplates due to degenerative changes may be widespread
**Bone marrow edema**	Present, not visible on conventional X-rays. Evident on MRI	Absent
**Paraspinous hematoma**	Often present, not visible on conventional X-rays. Evident on MRI	Absent
**Callus formation**	Typically present within the first 2–4 weeks. Then, callus is gradually removed	Absent
**Remodeling of bone**	Absent	Often present as residual findings following old, healed fracture

References: Ross and Moore, 2015 [42]*, *Alexandru et al., 2012 [40]*, *Panda et al., 2014 [41]

### Assessment of hip fractures

**Fig. 7:**
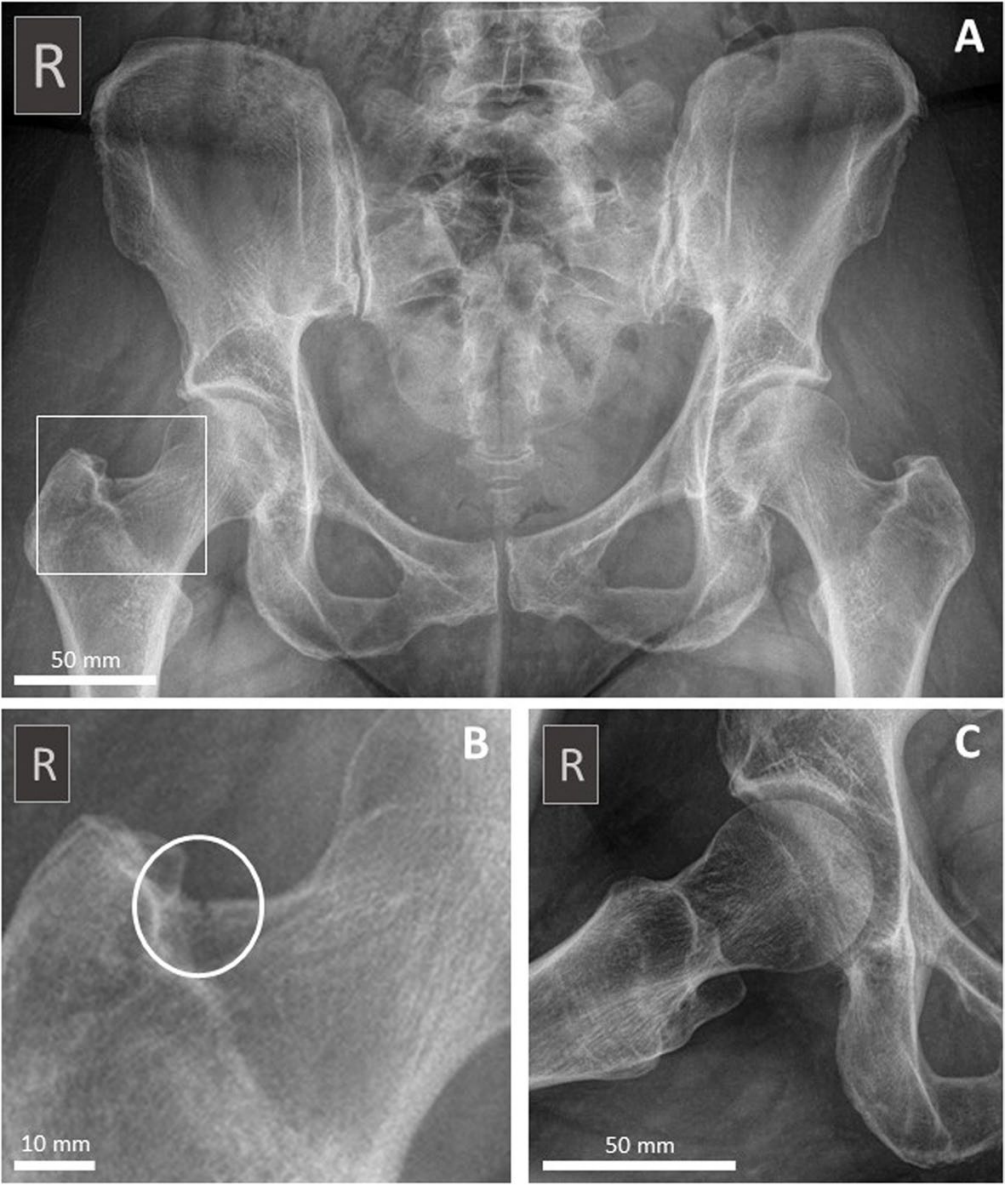
Discrete hip fracture on conventional X-rays. A 59-year-old female consulting a chiropractic clinic with idiopathic, ongoing debilitating right-sided groin pain through four weeks, gradually increasing severity despite intensive physiotherapy rehabilitation. An in-house standing X-ray examination of the pelvis (AP projection (**A**), closeup of the right hip from A (**B**) and frog leg view (**C**)) was performed showing generalized osteopenic appearance of the bone and a discrete fracture (white circle in **B**) through the cortex of upper part of the femoral neck on the right side. The patient was referred to the hospital where stabilizing surgery was performed. Medical treatment for DEXA-confirmed osteoporosis was initiated

The annual incidence of hip fractures in Denmark is among the highest in the world [43]. Patients with acute hip fractures rarely consult chiropractic practice due to the intense pain and lack of ambulatory function. However, when a hip fracture is osteoporotic in nature it may gradually develop/fail over a period of time until it finally fractures, which is somewhat similar to a stress fracture (Fig. 7) [44]. Although rare, these patients are more likely to consult the chiropractor prior to the final fracture and therefore pose a clinical challenge. In such cases, a thorough radiological evaluation may reveal osteopenia of the skeleton and sometimes discrete signs of a fracture (Fig. 7), but often the conventional radiographs are normal which makes referral based on clinical findings imperative [44, 45]. Osteoporotic fractures of the hip are most often located in the femoral neck or the intertrochanteric region of the proximal femoral shaft [46].

### Chiropractors’ practice

The majority of chiropractors utilize conventional radiological procedures for diagnostic imaging in clinical settings. The methods described in this paper makes it possible to identify patients with radiological signs of osteopenia and/or fracture (suspected osteoporotic in nature) at an early stage of osteoporosis. Hence, new or pre-existing X-rays (e.g. retrieved from other radiological hospital departments) should be evaluated in practice using the opportunistic approach described in the text. This involves, as a minimum, the high-risk patients (Table 1) who have risk factors for osteoporosis, e.g. elderly patient with low BMD, previous fracture in adulthood or loss of height [23]. In addition, we recommend that new or pre-existing X-rays (obtained for any reason) of patients above the age of 50 years, who are likely to receive relatively forceful energy transfer HVLA-SMT, should be examined with the procedures described.

We have outlined key features on conventional radiological images that raise suspicion of osteoporosis and should alert the chiropractor to refer a patient to the GP or if possible, directly for DEXA. Systematic and structured evidence-based descriptions of the radiological images and consistent transfer of important findings to the GP and/or relevant hospital department is crucial [7, 47]. This would ensure optimal utilization of the chiropractor’s radiological examination and interpretation. Accordingly, to assist further clinical evaluation, it is important that the chiropractors’ images are relevant, of highest quality, and well-described according to standards of care in the community. We are aware that there are differences between countries with regard to the permission of chiropractors to perform radiological evaluation. By Danish law, chiropractors in Denmark have full permission to conduct in-house radiological examination of patients. Furthermore, on clinical indications, they have permission to refer to hospital radiological examination including conventional radiology, MRI, and/or computed tomography (CT) scanning, and in some cases direct referral for DEXA is possible. An important part of this interdisciplinary agreement is the effective, and direct exchange of reports and images between chiropractors, GPs, and the hospital department through well-established encrypted electronic communication channels.

### Clinical implications

It is important to note that not all vertebral fractures have an osteoporotic etiology. Nonetheless, The Bone Health and Osteoporosis Foundation (BHOF) have stated that the identification of a vertebral fracture in an adult aged 50 years or above is diagnostic of osteoporosis, even in the absence of a BMD diagnosis [7]. Hence, when fractures are identified in patients following low-energy trauma, e.g. same-level falls, in patients with a history of previous fracture and/or in patients with predisposing medical conditions, osteoporosis should be suspected, and relevant examination instigated [7].

Early diagnosis of osteoporosis is important for initiation of relevant therapy and thereby reduction of the detrimental effects of the disease [6, 7]. In fact, the presence of an OVF significantly increases the risk of subsequent vertebral or other fractures, which may lead to a severe progressive clinical condition known as”the vertebral fractures cascade” [48]. Therefore, early identification of fractures could have significant clinical implications for the patients [6–8, 48]. Hence, suspicion of osteoporosis based on clinical findings and X-ray findings should alert the chiropractor to refer for relevant evaluation, including additional imaging [6, 7, 10].

It is important to emphasize that vertebral fractures may be present despite a normal GSQ-score (< 20% loss of height) [47]. Some fractures do not involve the anterior part of the vertebral body, and one study have suggested that osteoporotic vertebral fractures are more prone to exclusively involve the upper endplate [49]. On the other hand, some fractures, known as buckling, affect the anterior cortex only without depressing the upper endplate [49]. Even though GSQ and ABQ appear to be equally effective in identifying OVFs, ABQ seems to have a higher inter-rater reliability [39]. Overall, subtle structural changes do not necessarily score on the GSQ or the ABQ which sometimes makes determination of clinically relevant fractures difficult.

It is sometimes necessary to estimate the age of a vertebral fracture, for example, in cases where the cause of pain is uncertain or in insurance cases where the presence of a new fracture may have judicial consequences. In these cases, referral for MRI might be the ultimate option because, as described in Table 2, MRI will allow a more precise estimate of fracture age compared to a conventional X-ray exam [40, 41].

Osteoporotic fractures involving the hip may be difficult to detect clinically and radiologically in the initial phases. In cases where a hip fracture is suspected, referral to further diagnostic evaluation is warranted, irrespective of negative conventional radiological findings. In cases with significant clinical findings, direct referral to the emergency room (ER) must be effectuated.

### Recommendations

Based on this review, we give the following recommendations to the chiropractors’ conventional radiological examination for the purpose of contributing to early diagnosis of osteoporosis (Table 3).

**Table 3:** Recommendations for clinical practice

• Radiological examination should be based on clinical indications according to available guidelines
• Standardized protocols should be used for radiographic procedures
• Recently obtained images acquired for other purposes than osteoporosis can and should be used for vertebral fracture assessment when possible
• Radiological evaluation of patients above the age of 50 year should include an assessment of bone quality, GSQ, ABQ and SI, when applicable, irrespective of the purpose of the radiological examination
• Written referrals to the GP and/or specialized facilities should include a description of the findings and a conclusive diagnosis
• If a vertebral fracture is identified, the term “vertebral fracture” should be used
• Confirmed or suspected osteopenia, vertebral fracture, or hip fracture should always be referred to a medical facility for additional diagnostic evaluation
• The patient should be informed, and treatment may be offered according to guidelines

## Conclusions

This article presents an overview of evidence-based radiological procedures and recommendations that may promote early diagnosis of osteoporosis based on an opportunistic approach involving coincidental findings on X-rays. Our recommendations include adhering to clinical indications and guidelines and using standardized protocols for radiographic procedures. We highlight the value of evaluating the bone quality, the presence of hip and vertebral fractures and vertebral deformity using GSQ, ABQ and SI of all relevant X-rays obtained of patients above the age of 50 years, irrespective of the purpose for obtaining the X-rays. In addition, we stress the importance of providing detailed reports to relevant healthcare professionals when needed. We conclude that consistent, high-quality radiological procedures and evaluations from chiropractic radiological departments could feasibly contribute to the timely diagnosis of osteoporosis, ultimately minimizing the impact of osteoporosis-related complications on patients’ health.

## Data Availability

Not applicable.

## Abbreviations

ABQ: Algorithm-based qualitative method
AI: Artificial intelligence
ALARA: As-low-as-reasonably-achievable
AP: Anterior–posterior
BMD: Bone mineral density
BMI: Body mass index
DCNN: Deep Convolutional Neural Networks
DEXA: Dual-energy X-ray Absorptiometry Scanning
ER: Emergency room
GP: General practitioner
GSQ: Genant Semi-Quantitative tool
MRI: Magnetic resonance imaging
OVF: Osteoporotic vertebral fracture
SD: Standard deviations
SI: Singh index
VFA: Vertebral fracture assessment
WHO: World Health Organization
